# Effect of Surrounding Solvents on Interfacial Behavior of Gallium-Based Liquid Metal Droplets

**DOI:** 10.3390/ma15030706

**Published:** 2022-01-18

**Authors:** Ji-Hye Kim, Ye-Jin Park, Sooyoung Kim, Ju-Hee So, Hyung-Jun Koo

**Affiliations:** 1 Department of New Energy Engineering, Seoul National University of Science and Technology, 232 Gongneung-ro, Nowon-gu, Seoul 01811, Korea; gh5289@naver.com; 2 Department of Chemical and Biomolecular Engineering, Seoul National University of Science and Technology, 232 Gongneung-ro, Nowon-gu, Seoul 01811, Korea; pkpk22294@gmail.com; 3 Department of Chemical and Biomolecular Engineering, North Carolina State University, Raleigh, NC 27695, USA; skim94@ncsu.edu; 4 Material and Component Convergence R&D Department, Korea Institute of Industrial Technology, Ansan 15588, Korea

**Keywords:** liquid metal, interfacial behavior, solvents, impact dynamics, contact angles

## Abstract

Gallium-based liquid metal (GaLM) alloys have been extensively used in applications ranging from electronics to drug delivery systems. To broaden the understanding and applications of GaLMs, this paper discusses the interfacial behavior of eutectic gallium-indium liquid metal (EGaIn) droplets in various solvents. No significant difference in contact angles of EGaIn is observed regardless of the solvent types. However, the presence or absence of a conical tip on EGaIn droplets after dispensing could indirectly support that the interfacial energy of EGaIn is relatively low in non-polar solvents. Furthermore, in the impact experiments, the EGaIn droplet bounces off in the polar solvents of water and dimethyl sulfoxide (DMSO), whereas it spreads and adheres to the substrate in the non-polar solvents of hexane and benzene. Based on the dimensionless *We* number, it can be stated that the different impact behavior depending on the solvent types is closely related to the interfacial energy of EGaIn in each solvent. Finally, the contact angles and shapes of EGaIn droplets in aqueous buffer solutions with different pH values (4, 7, and 10) are compared. In the pH 10 buffer solution, the EGaIn droplet forms a spherical shape without the conical tip, representing the high surface energy. This is associated with the dissolution of the “interfacial energy-reducing” surface layer on EGaIn, which is supported by the enhanced concentration of gallium ion released from EGaIn in the buffer solution.

## 1. Introduction

Gallium-based liquid metal (GaLM) alloys in a liquid state at room temperature have attracted considerable attention because of their fluidity, low viscosity, low toxicity, high electrical/thermal conductivity, and deformability. When exposed to oxygen, a gallium oxide layer with a thickness of 1–3 nm forms on the GaLM surface, lowering the surface tension to ~0 [1,2,3,4]. Because of the low surface tension, the metals can be patterned into various structures, allowing GaLM to be used in flexible and stretchable electronics, strain or pressure sensors, and biomedical applications [2,5,6,7,8,9,10]. The metals have been handled and used in an inert gas environment for applications that require the oxide-free, metallic surface of GaLMs [11,12].

The surface of GaLMs is significantly affected by the type and composition of the liquid environment as well as the gas environment. For example, a gelatinous gallium oxide hydroxide (GaOOH) with lower mechanical strength than gallium oxide is formed in deionized (DI) water [13,14]. The gallium oxide skin is reduced in a strong acid or strong base, exposing the pure GaLM to the solvent [15,16]. In addition to aqueous solvents, the surface of GaLMs can also be manipulated when exposed to organic solvents. Lin et al., for example, reported a carbon layer-coated GaLM in ethanol [17]. Furthermore, organic solvents are also commonly used as dispersion media of GaLM micro-/nano-particles for applications of printable inks and electrolytes of energy devices [18,19,20]. To improve the understanding and applications of GaLMs, it is important to know the interfacial behavior of the metals in various solvents. However, to date, few studies on this topic have been conducted.

Herein, we present the interfacial behavior of a eutectic gallium-indium alloy (EGaIn, 75.5% Ga and 24.5% In by weight) on a Si wafer in various solvents, including polar (DI water, ethanol, dimethyl sulfoxide (DMSO)), nonpolar (hexane, benzene, silicone oil), and pH buffer solutions (pH 4, 7, 10). EGaIn is one of the most widely used GaLMs because, at room temperature, it is a homogeneous liquid at the eutectic composition. To reduce complexity, this study focuses on the interfacial characteristics of EGaIn, which is the gallium-indium binary alloy, as a model GaLM rather than gallium-indium-tin ternary alloy (i.e., Galinstan). To investigate the interfacial properties of EGaIn, we measured contact angles and conducted an impact experiment with EGaIn droplets in the various solvents. It was examined whether the presence or absence of the conical tip of EGaIn extruded from the syringe needle is determined by types of solvents with different dipole moments. Furthermore, the different impact behaviors of the EGaIn droplets were observed when they drop on the substrate in the solvents. Finally, the contact angles of the EGaIn droplets and the concentrations of the released ions in the pH buffer solutions (pH 4, 7, and 10) were measured to analyze how the acidic, neutral, and basic aqueous solvent environments affect the surface of the metal droplets, as well as their interfacial properties.

## 2. Materials and Methods

### 2.1. Materials

EGaIn (Gallium 75.5%, Indium 24.5%, >99.99%) and silicone oil (for oil bath, 100 mPa·s, product no. 85409) were purchased from Sigma Aldrich (St. Louis, MO, USA). DMSO (>99.5%), benzene (>99.5%), and n-hexane (>95%) were purchased from Daejung Chemicals & Metals (Siheung, Korea). Ethanol (>95.0%), and pH 4, 7, and 10 buffer solutions were obtained from Samchun Chemicals (Seoul, Korea). DI water (≥18 MΩ cm) was produced using a Millipore/Direct-Q3UV Water purification system (Burlington, MA, USA).

### 2.2. Measurement of Contact Angles and Impact Dynamics

The contact angles of EGaIn were measured using a drop shape analyzer (DSA100S, KRUSS, Germany) at 20 °C and analyzed using ImageJ (Java-based image processing program). A Si wafer was placed in a 5 × 5 × 5 cm^3^ cubic glass chamber and various solvents were poured into it. Subsequently, 4–5 μL of the EGaIn droplet was placed on the Si wafer through a 25 G syringe needle (outer diameter: 0.5 mm; inner diameter: 0.26 mm) in each solvent. For the impact dynamics analysis, EGaIn was dropped from a 22 G syringe needle (outer diameter: 0.72 mm; inner diameter: 0.41 mm) at 1 cm from the Si wafer immersed in each solvent at 20 °C. The impact behavior of the droplets was recorded using a Canon digital camera (Digital SLR Camera EOS 700D).

### 2.3. Elemental Analysis

Initially, 50 µL of the EGaIn droplet was placed in a vial filled with 3 mL of each pH buffer solution. The samples of the buffer solutions with the eluted ions were taken at given time points up to 24 h at 20 °C. The ion concentrations of the samples were measured using an inductively coupled plasma-mass spectrometer (iCAP-Q, Thermo Fisher Scientific, Waltham, WA, USA).

## 3. Results and Discussion

**Figure 1:**
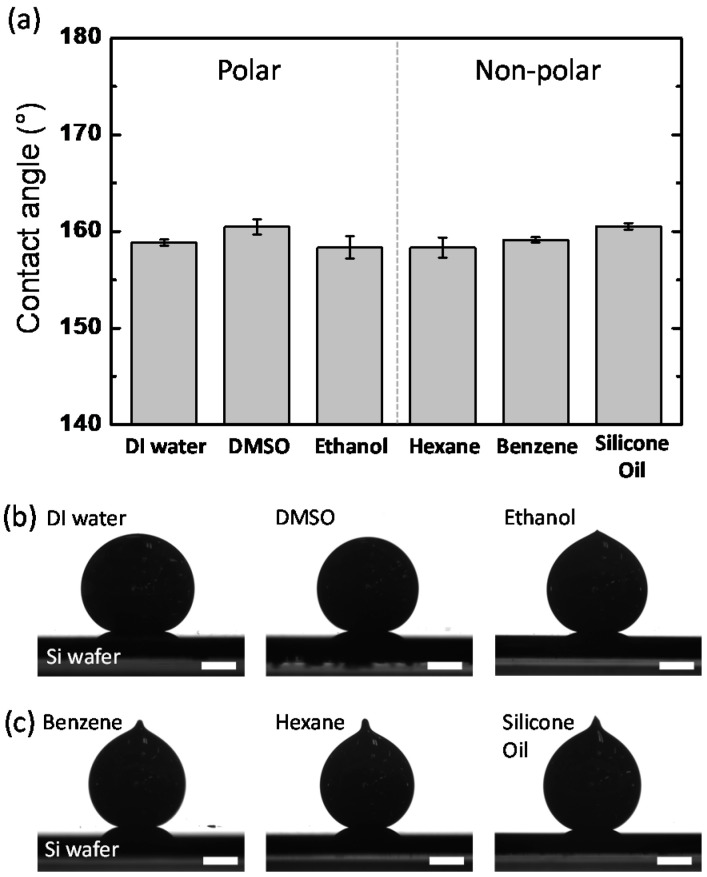
(**a**) Contact angles of EGaIn droplets on a Si wafer in polar and non-polar solvents. (**b**,**c**) Side-view images of EGaIn droplets placed on a Si wafer in (**b**) polar and (**c**) non-polar solvents. The scale bars are 0.5 mm. The contact angles were measured at 20 °C.

**Figure 2:**
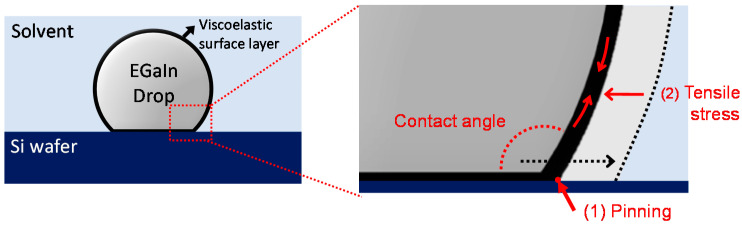
Schematic of the contact angle of the EGaIn droplet placed on a Si wafer. The viscoelastic surface layer (1) pins the metal to the substrate and (2) withstands the tensile stress.

As one of the most direct and intuitive approaches to identifying the interfacial energy, the contact angles of the EGaIn droplets were measured in different types of polar and non-polar solvents. In the case of the polar solvents, we selected water, DMSO, and ethanol. The values for their relative polarity are 1.000, 0.444, and 0.654, respectively [21]. We selected benzene, hexane, and silicone oil as non-polar solvents. Benzene and hexane have relative polarities of 0.111 and 0.009, respectively, and silicone oil has a low polarity [21,22,23]. Based on Young’s equation, the contact angle on an ideal flat solid surface is determined by the interfacial tensions of three immiscible phases [24]. Therefore, we hypothesized that the contact angles of the EGaIn droplets would vary depending on the polarity of solvents due to the different interfacial energy values between the solvents and EGaIn. Figure 1a shows the contact angles of the EGaIn droplets in various polar and non-polar solvents. Contrary to our expectation, all EGaIn droplets show high contact angles at ~160 °C with negligible difference regardless of the solvent types. This result may be associated with the viscoelastic thin layer newly formed on the EGaIn droplet in solvents. For EGaIn droplets in solvents, the surface of the metal could be coated with organic or inorganic layers such as oxides, hydroxides, self-assembled monolayers, and their composites, depending on the types of surrounding solvents [25,26,27]. It is known that gallium-rich surface layers formed on EGaIn readily adhere to a wide range of surfaces, including metals, metal oxides, plastics, and glass, as well as the native silicon oxide on the Si wafer. Consequently, when the EGaIn droplet is placed on the Si wafer, the surface layer adhering to the substrate (1) pins the EGaIn droplet to the contact line and (2) withstands the tensile stress to yield the surface layer (Figure 2) [28]. For this case, the contact angle behaves similarly to an advancing angle rather than a static contact angle. Thus, because of the sticky, solid-state surface layer formed on the EGaIn droplet in the solvents, it would be difficult to determine the interfacial energy of the EGaIn/solvents interface by the contact angle measurement.

Notably, the side-view images of the EGaIn droplets in the non-polar solvents differ from those in the polar solvents. In the polar solvents, except in ethanol, the EGaIn droplets have a spherical shape (Figure 1b), whereas in the non-polar solvents, the EGaIn droplets have conical tips on the top (Figure 1c). The conical tip forms when EGaIn bifurcates and is separated at the end of the needle by Plateau–Rayleigh instability [29] as the syringe needle is retracted. The presence or absence of the conical tip allows us to indirectly estimate the interfacial energy. The conical tip of the EGaIn droplet in the non-polar solvents in Figure 1c implies that the surface of the droplets has been modified to reduce the interfacial energy; otherwise, the droplets would form spherical shapes because of the strong cohesive force between the metal elements of EGaIn. The droplet in ethanol also has a little conical tip on the top presumably due to low interfacial energy compared to those in other polar solvents. Because the surface Ga atoms of EGaIn readily form Ga-O bonds [27,30], the polar solvents containing oxygen atoms may cover the droplet’s surface and cause it to behave similarly to a micelle. In contrast, in the non-polar solvents such as hexane and benzene, the surface of the EGaIn droplets is not likely to react with the solvent molecules to form Ga-C bonds [31]; however, it is still possible that the solvent molecules physically adsorb to the surface of the droplet. Thus, the interfacial energy of the EGaIn droplets in solvents is influenced by the types and polarity of solvents.

**Figure 3:**
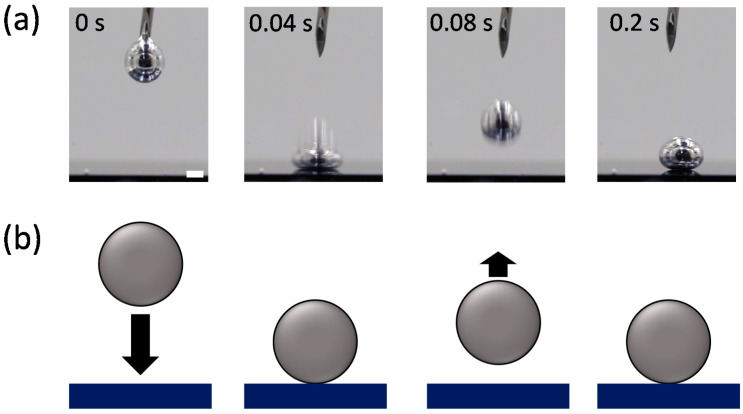
(**a**) Video snapshots of the EGaIn droplet impacting a Si wafer in water at 20 °C. Scale bar is 2 mm. (**b**) Schematic of the “bouncing” behavior of the EGaIn droplet in (**a**).

**Figure 4:**
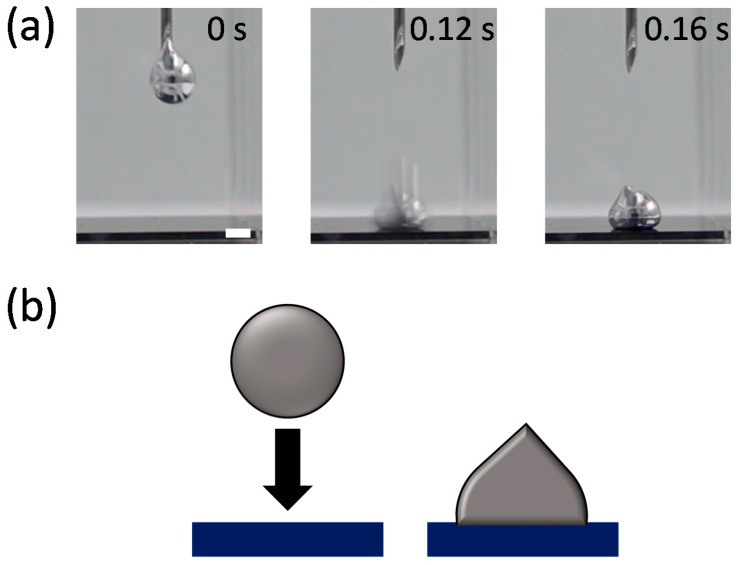
(**a**) Video snapshots of the EGaIn droplet impacting a Si wafer in hexane at 20 °C. Scale bar is 2 mm. (**b**) Schematic of the “adhesion” behavior of the EGaIn droplet in (**a**).

Analyzing the impact dynamics of droplets could be another way to examine the interfacial energy. The droplet impact dynamics, such as splash, bouncing, spreading, and adhesion, strongly depend on interfacial tensions as well as the impact velocity [32,33]. We observed the impact behavior of an EGaIn droplet falling on a Si wafer in the various solvents. Figure 3 and Figure 4 compare the impact behaviors of the EGaIn droplets in water (polar solvent) and hexane (non-polar solvent) when they collide with the Si wafer. In water, the EGaIn droplet bounces off after collision while maintaining its spherical shape. The droplet in another polar solvent of DMSO also shows similar bouncing behavior (Figure S1 in Supplementary Materials). In ethanol, the EGaIn droplet does not show significant bouncing behavior but slightly adheres to the wafer (Figure S1). The slight adhesion prevents the EGaIn droplet from sliding down on the Si wafer tilted by up to ~20°. (Figure S3). In contrast, in the non-polar solvents, hexane and benzene, the EGaIn droplets spread and adhere to the Si wafer upon collision (Figure 4 and Figure S2 in Supplementary Materials). In silicone oil, the EGaIn droplet neither bounces off nor adheres to the wafer. It gently lands on the wafer with high contact angle (Figure S2). The different adhesion behaviors of the EGaIn droplet in the non-polar solvents are observed when the wafer is tilted. In hexane and benzene, the EGaIn droplets adhere to the wafer and do not slide down even when the wafer is tilted by 22 ± 1°, whereas the droplet rolls off in silicone oil when the wafer is tilted only by 10° (Figure S3 in Supplementary Materials).

To explain the different impact dynamics of the EGaIn droplets depending on the solvent types, the Weber (*We*) number can be utilized. The *We* number represents the ratio of the kinetic energy on impact to the interfacial energy and is defined by the following equation:

(1) $$We=\frac{\rho v^{2}R_{0}}{\sigma }\;,$$

where *ρ* is the density of the solvent, *ν* is the velocity on impact, *R*_0_ is the droplet radius, and *σ* is the interfacial tension of the droplet. Based on the *We* number, the impact behavior can be predicted; when the *We* number is larger, the impacting liquid droplet is more likely to spread to the substrate [34,35]. In our impact experiment, the interfacial tension between the EGaIn droplet and the surrounding solvent is the most crucial parameter to determine the *We* number and therefore the impact dynamics of the EGaIn droplet as other parameters do not change significantly. Because the *We* number is inversely proportional to the interfacial tension, the bouncing behavior of the EGaIn droplet in water and DMSO, i.e., low *We* number, means relatively high interfacial tension of the liquid metal in the polar solvents. In contrast, the adhesion behavior of the EGaIn in hexane and benzene, i.e., high *We* number, indicates low interfacial tension of EGaIn in the non-polar solvents. In the additional impact experiment in the air (Figure S4 in Supplementary Materials), the EGaIn droplet spreads out and sticks to the Si wafer similarly as in hexane or benzene. It is well known that the surface tension of EGaIn in the air dramatically decreases due to the formation of the gallium oxide surface layer [1,2,3,4]. In ethanol, the EGaIn droplet slightly adheres to the Si wafer without the bouncing behavior. This may be because the interfacial tension of EGaIn in ethanol is not as high as that in other polar solvents. It has been reported that EGaIn in ethanol is coated by a carbon layer that could act as a surfactant to reduce the interfacial tension of EGaIn [17]. In silicone oil, the EGaIn does not spread to the Si wafer but maintains a high contact angle. This should be mainly because the impact velocity is low in the highly viscous oil medium (viscosity = 100 mPa·s), resulting in a decrease in the *We* number. Thus, the interfacial energy between EGaIn and various solvents can be examined by analyzing the impact dynamics of the EGaIn droplets in the solvents.

**Figure 5:**
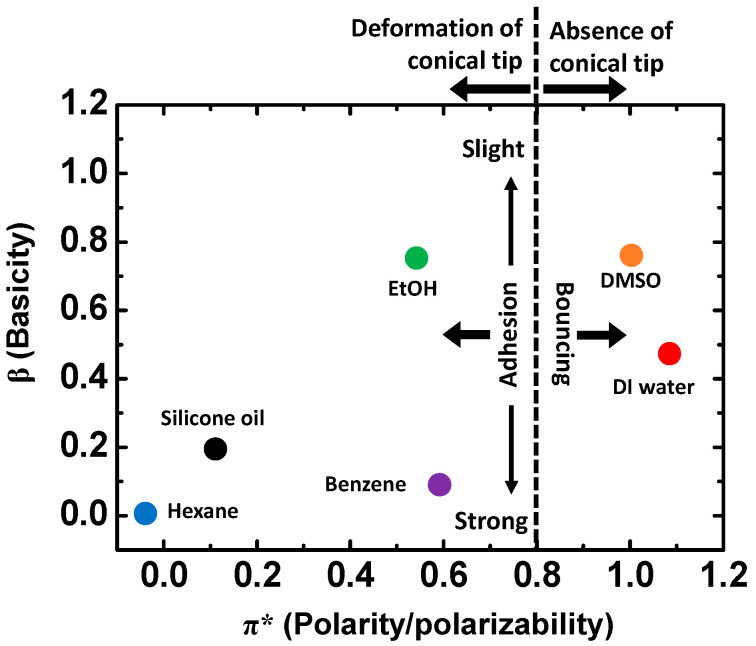
Interfacial behaviors depending on Kamlet–Taft parameters *β* and π*.

Table 1 compares the molecular structures and properties of the six polar and non-polar solvents and summarizes the experimental results of the interfacial characteristics of the EGaIn droplets in the solvents. The polar solvents, except for ethanol, have high surface tension, where the EGaIn droplet shows bouncing behavior on the Si wafer. The cohesive force between the solvent molecules is strong enough to prevent the droplet from adhering to the substrate and repel the droplet from the substrate. The ethanol has relatively low surface tension and presumably forms an interfacial layer, leading to the slight adhesion of EGaIn without any bouncing. The non-polar solvents tend to have low surface tension, where the EGaIn droplet spreads and adheres to the Si wafer. The silicone oil has extremely high viscosity compared to other solvents, which may prevent EGaIn from spreading and adhering to the wafer substrate. The interfacial behaviors of EGaIn can be correlated to Kamlet–Taft parameters, which include the information of basicity (*β*) and polarity (π*) of solvents (Table 1) [36]. Figure 5 shows the correlation between interfacial behavior of the EGaIn droplet and the Kamlet–Taft parameters. As *β* and π* increase, the EGaIn droplet tends to bounce off, whereas as *β* and π* decrease, it is more likely to show strong adhesion to the substrate. Thus, the interfacial characteristics of EGaIn strongly depend on the types and surface tension values of surrounding solvents.

**Figure 6:**
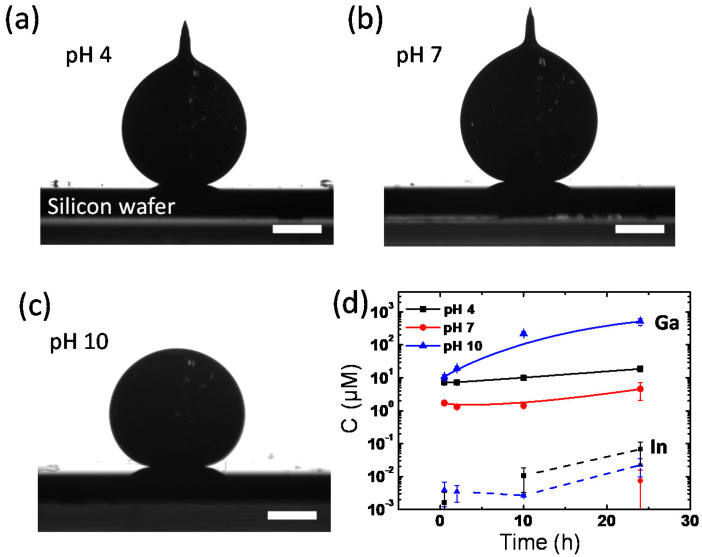
Side-view images of EGaIn droplets placed on a Si wafer in (**a**) pH 4, (**b**) pH 7. and (**c**) pH 10 buffer solutions at 20 °C. Scale bars are 0.5 mm. (**d**) Time-dependent Ga and In ion concentrations dissolved into buffer solutions from the EGaIn liquid metal droplet.

In an aqueous environment, pH values could also be critical in determining the interfacial properties of EGaIn because, in strong acids or bases, the gallium-rich surface layer (e.g., gallium oxide and gallium oxide hydroxide) on EGaIn is susceptible to dissolution. We observed the EGaIn droplet in different pH solutions to understand how the pH value affects the interfacial properties of the EGaIn liquid metal in the aqueous environment. Figure 6a–c shows the contact angles of EGaIn droplets in pH 4, 7, and 10 buffer solutions. The droplets in all of the pH buffer solutions have high contact angles of ~160°, which is comparable to the values previously discussed in Figure 1a. The shapes of the droplets vary depending on the pH of the buffer solutions. In pH 4 and 7 buffers, the EGaIn droplets form the conical tips on top, whereas in pH 10 buffer solution, the EGaIn droplet forms a spherical shape. This is because the surface layer of EGaIn droplet is dissolved in the strong basic condition of pH 10, whereas the acidity of the pH 4 buffer solution is not strong enough to remove the surface layer thoroughly. As per the Pourbaix diagram, the gallium oxide skin of EGaIn is reduced at pH < 3 or pH > 10 [15,16]. Interestingly, the EGaIn droplet in the pH 7 buffer solution has the conical tip, which differs from that of the tip-less spherical EGaIn droplet in DI water (Figure 1b), although the DI water should also have a near-neutral pH. We speculate that this may be due to the ions dissolved in the buffer solutions. A large amount of potassium, sodium, or phosphate ions might lower the interfacial energy between EGaIn and the aqueous buffer solution.

To confirm the dissolution of the surface layer by the reduction in the pH 10 buffer solution, the amount of Ga and In ions in each pH buffer solution in Figure 6a–c was measured and compared using mass spectroscopy. The time-dependent ion concentrations of Ga and In dissolved from EGaIn droplets in each pH buffer solution are shown in Figure 6d. In all pH conditions, Ga ions are more abundantly released from EGaIn than In ions, indicating that the surface layer is gallium-rich. Both concentrations of Ga and In ions increase with time but differ with pH. The elution of Ga ions from EGaIn occurs most at pH 10 and the least at pH 7. In the pH 10 buffer solution, Ga ions eluted are up to 688 μM in 24 h, which is 112 times higher than the concentration of Ga ions eluted in the pH 7 solution. It is caused by the basic solution reducing gallium oxide to Ga ions. Thus, in an aqueous environment, the pH value is another crucial factor to determine the interfacial energy of EGaIn because of the pH-dependent elution of the gallium-rich surface layer.

## 4. Conclusions

To summarize, we investigated the interfacial characteristics of EGaIn droplets in different types of polar and non-polar solvents. The difference in contact angles of the EGaIn droplets was insignificant, and the conical tip on top of the EGaIn droplets was formed in solvents with low surface tension. The impact dynamics of the EGaIn droplets in various solvents were analyzed where the droplet showed the different impact behaviors of bouncing, sitting, and adhesion after collision with the Si wafer. With the dimensionless *We* number, the different impact behaviors could be explained by the interfacial tension values of the EGaIn droplets in each solvent. In general, EGaIn tends to bounce off in polar solvents such as water and DMSO, representing high interfacial energy. In contrast, EGaIn spreads and adheres to the substrate in the non-polar solvents, indicating low interfacial energy. Furthermore, to investigate the pH effect on the interfacial behavior of EGaIn, the contact angles and shapes of the EGaIn droplets in pH 4, 7, and 10 buffer solutions were compared. The EGaIn droplet maintained the conical tip on top in pH 4 and 7, whereas the metal droplet had a spherical shape in pH 10 solution. This is presumably due to the pH-dependent dissolution of the surface layer, which was supported by the measurement of the ions released from EGaIn in the buffer solutions. This research provides a useful guideline for selecting suitable solvents for using GaLMs depending on their fabrication processes and applications. Further study is now underway to investigate the chemical compositions and molecular structures of the surface layers formed on EGaIn surface in various solvents.

## Figures and Tables

**Table 1:** Molecular structures and physical properties of solvents used and interfacial characteristics of EGaIn in the corresponding solvents.

	Polar Solvents			Non-Polar Solvents		
	DI Water	DMSO	Ethanol	Hexane	Benzene	Silicone Oil
Molecular structure	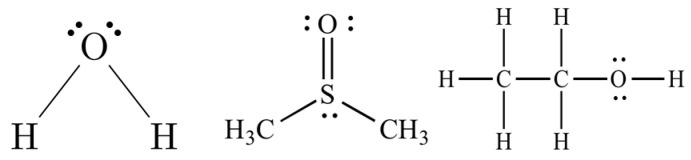			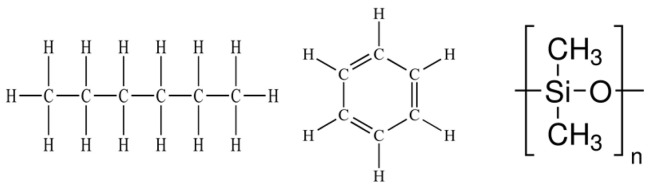		
Relative polarity	1	0.444	0.654	0.009	0.111	-
Dipole moment (D)	1.8	3.72	1.66	0	0	0.6–0.9
Hydrogen bond acceptor (*β*)	0.47	0.75	0.76	0.00	0.10	0.19^1^
Dipolarity-polarizability (π*)	1.09	1.00	0.54	−0.04	0.59	0.11 ^1^
Surface tension (mN/m) in air (25 °C)	72	43.5	22.3	18.8	28.9	<16
Viscosity (mPa·s) (25 °C)	0.89	1.99	1.04	0.30	0.603	100 (20 °C)
Interfacial characteristics of the EGaIn droplets in corresponding solvents						
Contact angle on Si wafer (°)	158	160	158	158	159	160
Impact behavior	Bouncing	Bouncing	Slight adhesion	Adhesion	Adhesion	Sitting ^2^
Formation of conical tip after dropping	×	×	△	O	O	O

^1^ The values of silicone oil were derived from poly(dimethyl siloxane); ^2^ Neither bouncing nor adhesion, but sitting with a high surface tension.

## Data Availability

Not applicable.

## Supplementary Materials

The following supporting information can be downloaded at: https://www.mdpi.com/article/10.3390/ma15030706/s1, Figure S1: Video snapshots of a EGaIn droplet impacting on a Si wafer (a) in DMSO and (b) in ethanol. Scale bars are 2 mm. The red arrows indicate the movement of EGaIn droplets; Figure S2: Video snapshots of a EGaIn droplet impacting on a Si wafer in (a) benzene and (b) silicone oil. Scale bars are 2 mm; Figure S3: Video snapshots of EGaIn droplets when the wafer is tilted in (a) ethanol, (b) benzene, (c) hexane, and (d) silicone oil. Scale bars are 2 mm. The tilting angles are 22 ± 1° in (a–c) and 10° in (d); Figure S4. Video snapshots of a EGaIn droplet impacting on a Si wafer in the air. Scale bar is 2 mm.
