# Supplementary material for: Effect of Surrounding Solvents on Interfacial Behavior of Gallium-Based Liquid Metal Droplets
Source: Materials (Basel). 2022 Jan 18;15(3):706. doi: 10.3390/ma15030706 (PMC8837161; doi:10.3390/ma15030706)
Supplement: Supplementary file 1 [file materials-15-00706-s001.zip › materials-1556609-supplementary.pdf]

*materials*

MDPI

*Article*

# Effect of Surrounding Solvents on Interfacial Behavior of Gallium-Based Liquid Metal Droplets

**Ji-Hye Kim [1,†], Ye-Jin Park [2,†], Sooyoung Kim [3], Ju-Hee So [4,*] and Hyung-Jun Koo [2,*]**

1 Department of New Energy Engineering, Seoul National University of Science and Technology, 232 Gongneung-ro, Nowon-gu, Seoul 01811, Korea; gh5289@naver.com
2 Department of Chemical and Biomolecular Engineering, Seoul National University of Science and Technology, 232 Gongneung-ro, Nowon-gu, Seoul 01811, Korea
3 Department of Chemical and Biomolecular Engineering, North Carolina State University, 27695 Raleigh, NC, USA
4 Material and Component Convergence R&D Department, Korea Institute of Industrial Technology, Ansan 15588, Korea
* Correspondence: jso@kitech.re.kr (J.-H.S.); hjkoo@seoultech.ac.kr (H.-J.K.)
† These authors contributed equally to this work.

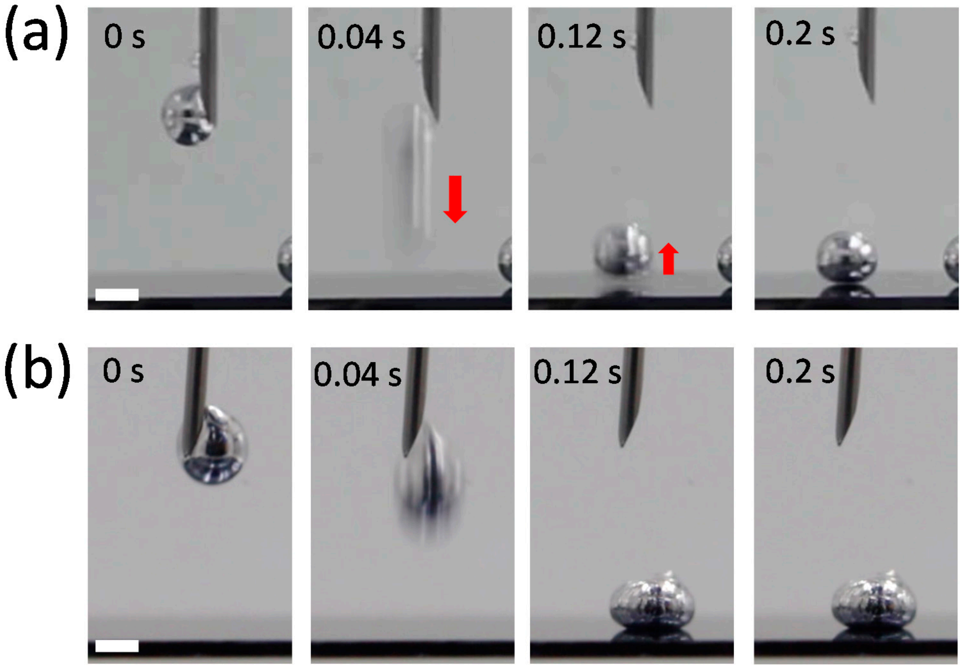


**Figure S1.** Video snapshots of a EGaIn droplet impacting on a Si wafer (a) in DMSO and (b) in ethanol. Scale bars are 2 mm. The red arrows indicate the movement of EGaIn droplets.

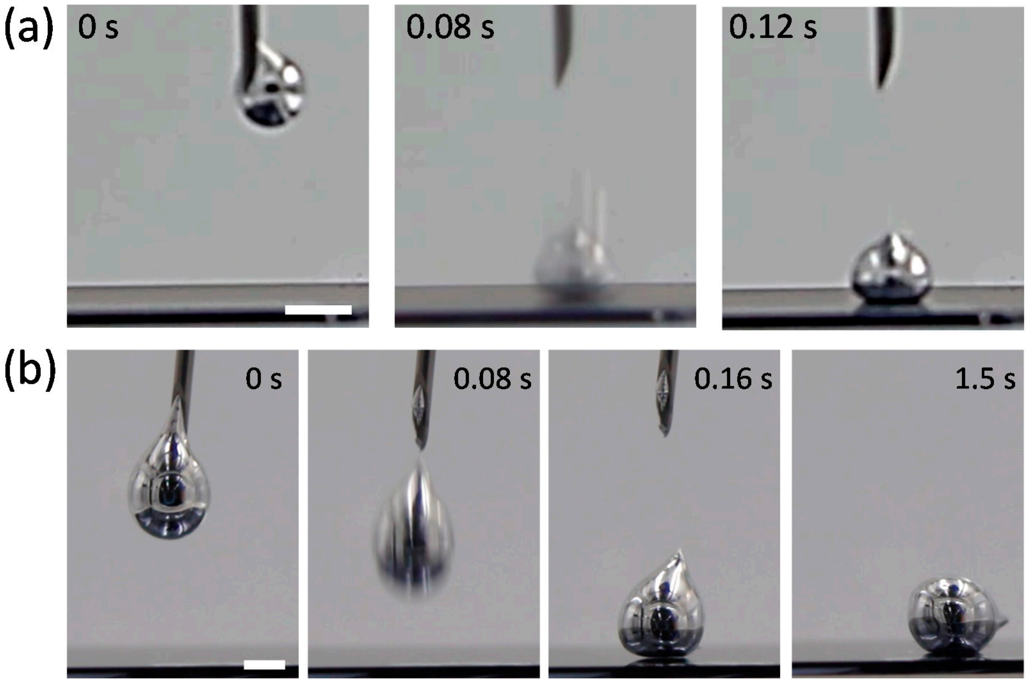


**Figure S2.** Video snapshots of a EGaIn droplet impacting on a Si wafer in (a) benzene and (b) silicone oil. Scale bars are 2 mm.

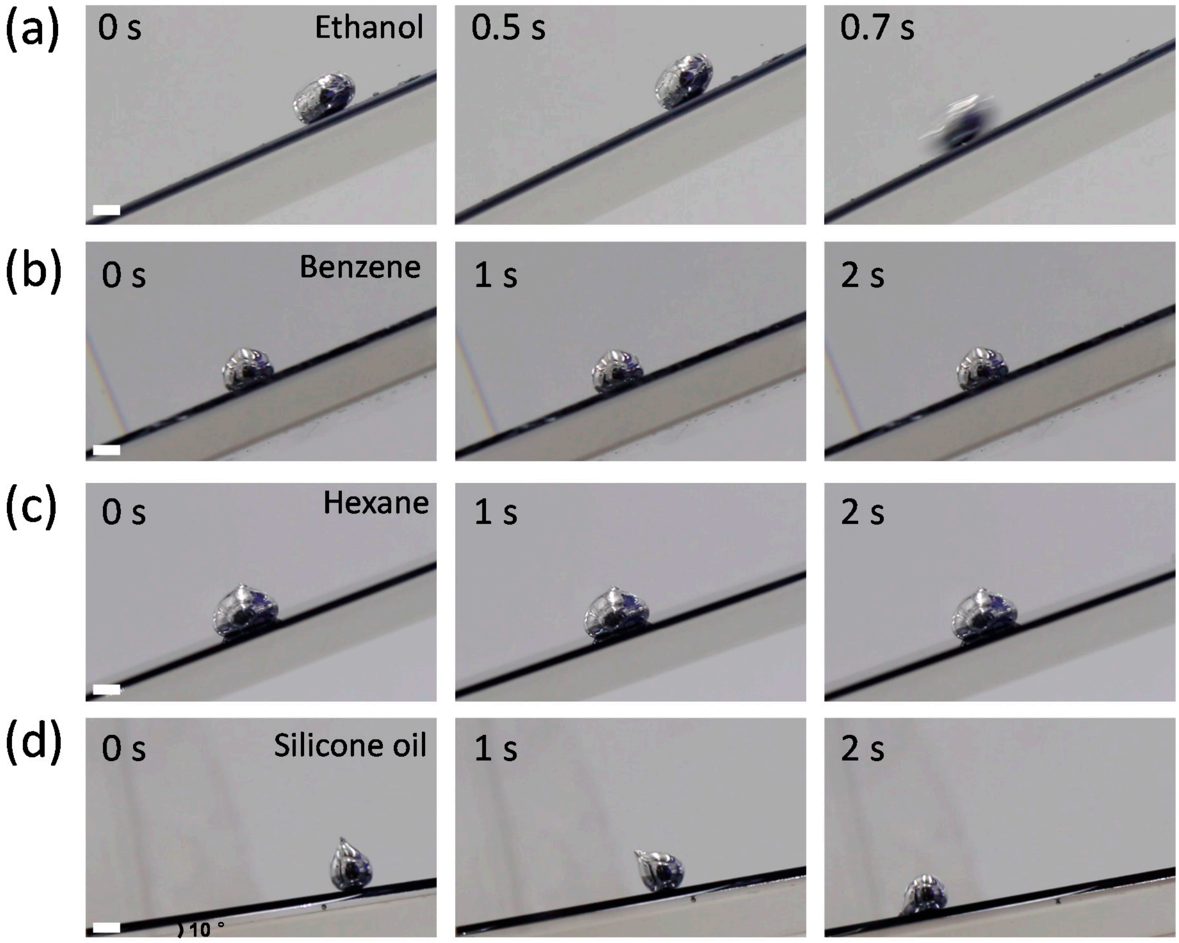


**Figure S3.** Video snapshots of EGaIn droplets when the wafer is tilted in (a) ethanol, (b) benzene, (c) hexane, and (d) silicone oil. Scale bars are 2 mm. The tilting angles are 22 ± 1° in (a)-(c) and 10° in (d).

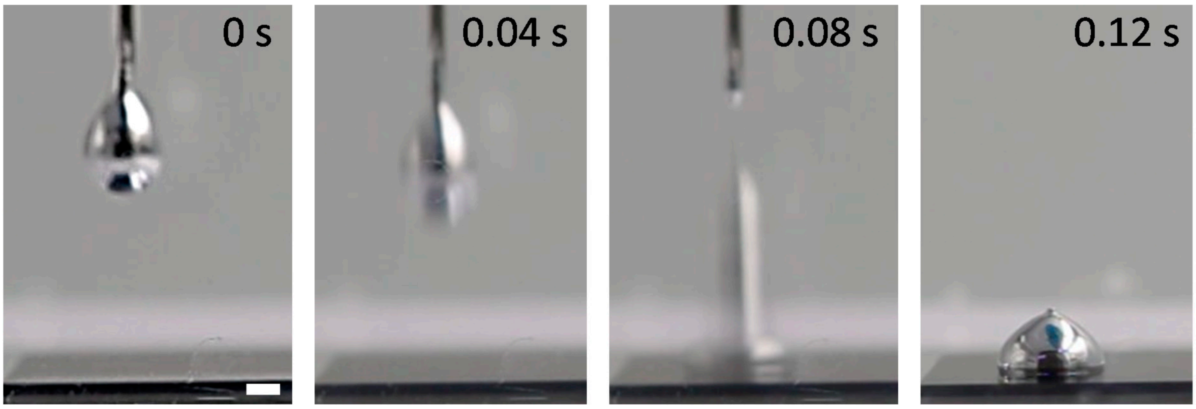


**Figure S4.** Video snapshots of a EGaIn droplet impacting on a Si wafer in the air. Scale bar is 2 mm.
